# Nutrient and Trace Elements in Suburban Sugar Maple (*Acer saccharum*) Sap, Syrup, and Soils from Massachusetts, Connecticut, New Jersey, and Pennsylvania

**DOI:** 10.1007/s00128-025-04055-4

**Published:** 2025-05-10

**Authors:** Justin B. Richardson, Eric Vukicevich, Eric C. Sirkovich

**Affiliations:** 1 Department of Environmental Sciences, University of Virginia, Charlottesville, VA 22904 USA; 2 Environmental Sciences Program, University of Massachusetts Amherst, Amherst, MA 01003 USA; 3 Botany Department, Connecticut College, New London, CT 06320 USA

**Keywords:** Sugar maple, Urban agriculture, Urban forestry, Toxic elements, Total hazard quotient

## Abstract

Production of maple syrup from sugar maples (*Acer saccharum*) in suburban areas lies at the intersection of urban farming and forestry, providing an artisanal food as well as ecosystem services. However, urban areas can be enriched with trace elements due to industrial, agricultural, and municipal pollution, which can potentially affect sap and syrup chemistry. Here, we collected soils, sap, and maple syrup from four artisanal maple syrup producers in four suburban areas across the northeastern United States to assess nutrient and trace element concentrations. Soil As and Pb concentration approached or exceeded EPA limits while Cd, Cu, and Zn were far below EPA limits. Sap and syrup As, Cd, Ni, Pb, and Zn concentrations reached or exceeded FDA limits for food. However, Total Hazard Quotients suggest that urban maple syrup consumption poses low to no health risk to adults and children.

## Introduction

Urban farming and forestry initiatives have increased in many of the metropolitan areas around the United States and globally (Palmer 2018; Urban and Community Forestry Program 2016) as ways to increase fresh food access and food sovereignty, decrease reliance on industrial agriculture, along with many other societal and ecosystem services provided by greenspaces and urban trees. Urban farming and forestry practices are often borne from a grassroots framework, which can lack access to equipment and testing that act as safeguards against environmental contaminants that can make their way into food via soil (Wortman and Lovell 2013). Urban soils have received trace element pollution from decades of industrial pollution (e.g. smelting, coal combustion, metal refining), inorganic pesticides (e.g. lead-arsenate), waste incineration, and automobiles (e.g. leaded-gasoline, metal particulates) (Paltseva et al. 2022). Unlike organic contaminants that can be degraded or transformed by microbial or physicochemical processes, metal contaminants can reside for decades to centuries.

Sugar maple (*Acer saccharum*) is an important tree across the northeastern United States economically, culturally, and ecologically. Sugar maple is the dominant tree for syrup production commercially and historically used by Native Americans (Perkins and van der Berg, 2009) and is the official state tree for New York, Vermont, West Virginia, and Wisconsin. Production of syrup in urban, suburban, and peri-urban areas as an artisanal food has received little study. The present study aims to bring a food safety and environmental toxicology lens more often seen in urban agriculture studies to an urban forestry product. Maple syrup production stages include (1) tapping and collection of sap flow from maple trees during early spring, (2) filtering the sap for particulates, and (3) evaporation to condense the sugar content by removing moisture (see full process description by Perkins and van den Berg 2009; Saraiva et al. 2022). Contamination of maple syrup can include using partially oxidized metal taps, buckets, evaporation pans, or bronze gear pumps (Northeast Forest Experiment Station 1982; Robinson et al. 1989; Saraiva et al. 2022). Sugar maple has relatively high Ca and P requirements and can accumulate other similarly sized divalent cations such as Mn, Pb, and Zn (St. Clair et al. 2008; Saraiva et al. 2022). Due to the grassroots nature of artisanal maple syrup production, it is unknown if tapping these trees poses any risks due to toxic element accumulation.

The overarching goal of this study was to investigate trace elements in sugar maple sap and syrup produced in suburban areas across the northeastern United States and consider if soils drive uptake rates and if exposure rates may be hazardous to children and adults. Due to the artisanal nature of local production of maple syrup by land-owners, potential exposure escapes regulatory agencies and practitioners may not consider testing the product for potentially toxic element concentrations. The specific objectives of this study were to: (1) evaluate maple sap and syrup for potentially toxic trace elements, (2) determine if soil concentrations of nutrients and potentially toxic elements relate to sap and syrup concentrations, and (3) evaluate risk from maple syrup trace element concentrations for consumption by adults and children. The results of this study shall provide insight on safety of maple syrup for artisanal production and consumption.

## Methods

### Study Sites, Sap and Syrup Collection and Processing

Sampling occurred in Spring 2022 for Amherst, MA and West Hartford, CT and in Spring 2024 for Roselle, NJ and West Scranton, PA. These areas are suburban cities and towns within the metropolitan area of larger cities (> 70,000 residents in population) and were studied to capture a range of socioeconomic conditions, ethnic composition, and anthropogenic influences. At each suburb, ongoing artisanal maple syrup production operations were identified and sap was collected and boiled down to generate different batches of maple syrup at each city (Table 1). Trees were all > 25 cm in diameter at breast height. Sap is the unaltered form that is collected from the trees during spring and syrup is produced from evaporating the sap and removal or separation from bottom sediments and floating debris. Sap was collected in mixed metal and food-grade plastic collectors at each production. For the digestion of sap and syrup, a 0.5 g subsample was first treated with 10 mL of 30% H_2_O_2_ to remove as much sugar as possible and then treated with 3 mL of reverse aqua regia (9:1 trace metal grade 15.7 M HNO_3_ + 12 M HCl) and heated at 70˚C for 60 min in closed tubes. The sap and syrup digests were diluted to 50 mL using de-ionized water. For every 10 samples, a preparation blank and corn syrup material spiked with Inorganic Ventures multielement standard 71-A were included.

**Table 1:** Number of samples collected at the four suburban artisanal maple syrup production operations

Suburb studied	Soils collected	Trees sap collected	Sap collection dates	Syrups studied
Amherst, MA	18	6	April 2022	3
Roselle, NJ	12	18	March 2023	4
Scranton, PA	12	8	April 2023	4
West Hartford, CT	12	27	April 2022	5

### Soil Collection, Processing, and Digestion

Mineral soil samples were collected at a distance of 1–2 m from the maple trees tapped for syrup. For the collection, surface materials (e.g. grass, other plants, mulch, etc.) were removed. An auger was used to collect approximately 0.5 kg of soil per sample from a depth of 0 to 20 cm below the surface. The soil samples were oven-dried at 60 °C for 48 h, sieved to < 2 mm, and homogenized. %Sand and %Clay were determined using a modified Bouyoucos hydrometer method and %SOM was determined using loss-on-ignition. A strong acid digestion following US EPA method 3050B was used to quantify the pseudototal fraction of elements. The soil was subsequently digested with 5 mL of 9:1 ratio of trace metal grade nitric acid to hydrochloric acid (15 M HNO_3_ + 10 M HCl, Fisher Scientific) and heated to 70 °C for 45 min using a hot plate. The digest was allowed to cool and diluted to 50 mL using 18.2 MΩ cm deionized water. For every 20 samples, a preparation blank and standard reference material (NIST 2709a San Joaquin Soil) were included. The maple sap, maple syrup, and soil digestions were analyzed for macroelements (Al, Ca, K, Mg, Na, Fe) using an Agilent 5110 Inductively Coupled Plasma– Optical Emission Spectrometer (Agilent Technologies, Santa Clara, CA, USA) and trace elements (As, Cd, Co, Cr, Cu, Ni, Pb, Sb, Se, Sn, Th, U, Zn) using an Agilent 7700x Inductively Coupled Plasma Mass Spectrometer (ICP-MS). Standard Reference Material recovery rates for NIST 2709a San Joaquin soil were 81 to 113% of their certified values and corn syrup spike recoveries were 82 to 109%. Digestion procedural blanks were < 0.01 ng/g for all trace elements. Duplicates were within < 4% coefficient of variation (CV) for soils and < 11% CV for saps and syrups. Limits of detection were 0.01 ng/g for trace elements.

### Data Analyses and THQ Calculations

Average values are presented in text and in figures ± 1 standard error and an alpha of 0.05 was used for significance. Descriptive statistics, nonparametric statistical tests, and linear regressions were calculated in MATLAB R2023a (Mathworks, Natick, MA, USA). Target hazard quotients (THQs) were used to determine the non-carcinogenic risk to human health from trace elements (As, Cd, Cr, Cu, Ni, Pb) during consumption. THQ was calculated using the following variables: Cte is the trace element concentration in maple syrup (mg/kg wet weight); EF is the exposure frequency (180 d/yr); IR is the ingestion rate (maple syrup consumption rate) to match the 2021 US per capita consumption rate of syrups by Americans (USDA Economic Research Service’s Food Availability Data); averaging time AT is the average time of consumption in days per year (365 days). Three body weights (BWs) were used: average body weight of a US adult of 80 kg, larger child (grade schooler) of 40 kg, and smaller child (toddler) of 10 kg. ED is the exposure duration and for the adult was the United States average life expectancy (77 years), for the small child is 2 years and for larger child is 6 years. Long-term exposure duration LT is equal to exposure duration. Maple syrup IRs were estimated to be 8 to 102 g/d for adults, 8 to 90 g/d for larger child, and 0.5 to 8 g/d for smaller child. These were estimated using annual US per capita maple syrup consumption rate (g/d) for adults as a baseline with higher rates estimated as portions of adult sugar consumption rates (> 20 years of age), adolescence (6 to 19 years of age), and toddler (1 to 2 years of age) daily sugar consumption rates from Drewnowski and Rehm (2014) and Herrick et al. (2020).

RfD is the oral reference dose for each individual trace element. RfD values ([mg/kg]•d) used were: 0.0003 for As, 0.001 for Cd, 0.003 for Cr, 0.04 for Cu, 0.02 for Ni, and 0.002 for Pb. Reference oral doses set for trace elements by the US EPA were used for THQ calculations. The THQ was calculated using a Monte Carlo Simulation in MATLAB R2023a for each site-specific CTe and IR rates randomly chosen between the range of consumptions for adults, larger child, and smaller child using Eq. 1. The variables EF, ED, AT, LT, and BW were held constant for each age group, trace element, and city or town and ran for 5000 iterations.

1 $${\text{THQ}} = ({\text{Cte}} \cdot {\text{EF}} \cdot {\text{ED}} \cdot {\text{IR}})/({\text{AT}}\cdot{\text{LT}} \cdot {\text{BW}} \cdot {\text{RfD}})$$

If individual or summed THQ values were determined to be < 1, it can be assumed to pose negligible increase in non-carcinogenic human health risk to an individual over their exposure while THQ values > 1 pose a non-carcinogenic risk to consumers.

## Results and Discussion

### Suburban Soil Concentrations

**Fig. 1:**
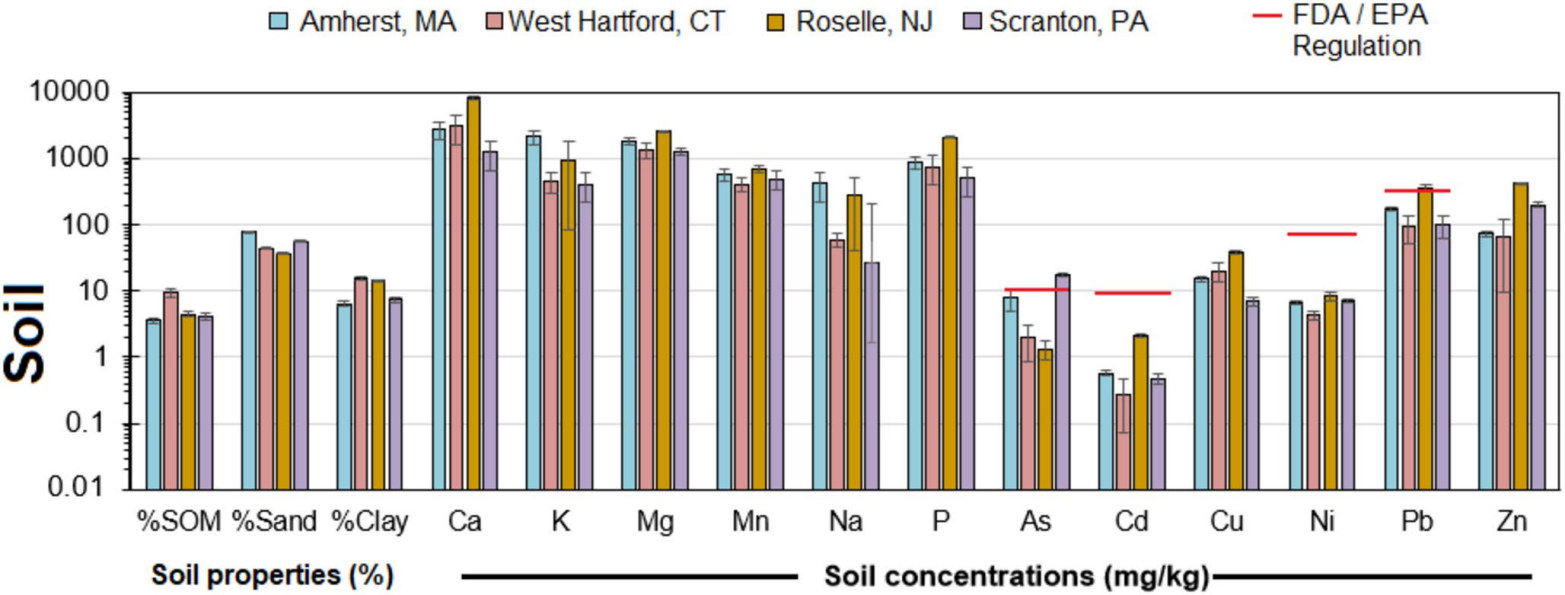
Nutrient and trace element concentrations and soil properties in studied suburban soils. Bars represent the mean concentration with standard error bars present. The red line denotes the US EPA regulatory limit for soils

Soil nutrient concentrations (Fig. 1) were within typically observed strong acid digestions for rural and urban forest soils in the regio (Smith et al. 2014). However, there were significant differences among the four towns. Soil K and Na were significantly higher in Amherst, MA than the other towns. Roselle, NJ had significantly higher Ca, Mg, and P than the other towns. Soil Cr, Cu, and Ni concentrations were within the range typically observed by strong acid digestion for rural forest and agricultural soils. Soil As concentrations in Amherst, MA and West Scranton, PA wereelevated compared to concentrations found across the eastern US of 5.4 ± 3.3 mg/kg (Smith et al. 2014). Amherst, MA and Roselle, NJ had elevated concentrations of Pb compared to values across the eastern US of 29 ± 20 mg/kg (Clark and Knudsen 2013; Smith et al. 2014). Lastly, Roselle, NJ had elevated soil Cd and Zn concentrations compared to values across the eastern US of 0.3 ± 0.2 mg/kg for Cd and 54 ± 39 mg/kg for Zn (Smith et al. 2014). Soil pH comparable across all four cities from 5.2 to 5.9 while %SOM was significantly greater for Roselle NJ at 9% than the other three cities of 3.8% at Amherst MA to 4.5% at Scranton PA. Across all soils and cities, we found that soil Cd, Cr, P, Pb, and Zn concentrations and %SOM and %Clay were all significantly correlated with each other (R^2^ > 0.25,*p* < 0.05). Soil concentrations of As, Al, Ca, Cu, K, Mg, Ni, soil pH, and %Sand were generally not correlated or had one or two significant correlations with other soil parameters.

Soil As concentrations in Amherst, MA were elevated likely due to historical domestic and municipal uses of arsenate pesticides. The high sand content and low %SOM at Amherst, MA suggest sorption is unlikely promoting the retention and accumulation of As. The elevated Cd, Pb, and Zn concentrations at Roselle, NJ could be due to similar sourcing industrial manufacturing, vehicle emissions, and waste incineration that have occurred across the New York City-Newark metropolitan area (Clark and Knudsen 2013; Paltseva et al. 2022) or be elevated due to higher SOM and clay sorption capacity in their soils. West Scranton, PA had significantly higher soil As concentrations than the other towns, which was unexpected, as typically other elements are associated with iron works and coal combustion such as Cu, Mn, Pb and Zn. West Hartford, CT had the lowest soil Cd, Cr, Ni, and Zn concentrations which was expected as it is a more recently settled, wealthy suburb that has not served as an industrial area in the 1900s (Dougherty 2021). Lastly, we compared soil concentrations with EPA regulatory limits. Soil Cd, Cr, Cu, and Zn were far below regulatory limits, showing that our study areas generally represent typical suburban soils. However, soil As concentrations exceeded EPA regulatory limits in samples from Amherst, MA and West Scranton, PA. Moreover, soil Pb concentrations exceeded EPA regulatory limits in samples from Roselle, NJ.

### Maple Sap

Maple sap concentrations of Al, Ca, K, Mg, Na, and P were in the upper range or exceeded concentrations measured in commercial maple sap reported by Perkins and van den Berg (2009) andConquer et al. (2023). Maple sap and adjacent soil nutrient concentrations were evaluated for linear relationships and only K and P were significantly correlated between sap and soil concentrations (R^2^ > 0.35,*p* < 0.01), agreeing with Conquer et al. (2023). The elevated nutrient concentrations may be due to the enhanced concentrations from sugar maple trees accessing anthropogenic nutrients. Now considering trace elements in maple sap, only sap Cu concentrations were within the FDA food/EPA drinking water regulatory limits for all sites. Maple sap As and Pb concentrations exceeded FDA/EPA regulatory limits for three suburban towns, but not for Roselle, NJ. Maple sap Cd and Zn concentrations exceeded FDA/EPA limits at West Scranton, PA. Maple sap Ni concentrations exceeded FDA/EPA limits for only Amherst, MA, which may be due to the use of galvanized metal sap collection buckets, which were not used at the three other syrup production operations.

Examining potentially toxic elements among towns, Amherst, MA and West Scranton, PA generally had higher maple sap As, Cd, Ni, Pb, and Zn concentrations than the other two towns. Maple sap and adjacent soil As, Cd, Cr, Cu, Ni, Pb, and Zn concentrations were evaluated for linear relationships and were not significantly correlated (R^2^ < 0.20, *p* > 0.05). When considering that elevated soil concentrations occurred at Roselle, NJ, these results suggest that soil concentrations are not the dominant factor controlling metal uptake by the sugar maple trees. The agrees with Conquering et al. (2023) that found sap Cd, Pb, and Zn concentrations can be decreased with higher Ca abundance in the soil. Maple sap Cu concentrations were below FDA/EPA regulatory limits and showed the opposite pattern as the other metals with higher concentrations occurring for West Hartford, CT and Roselle, NJ. This was an unexpected pattern, that the suburban town soils with the highest and lowest soil concentrations wouldproduce comparablesap concentrations. This highlights that soil tests alone cannot predict uptake rates of trace elements by sugar maple trees.

### Maple Syrup

**Fig. 2:**
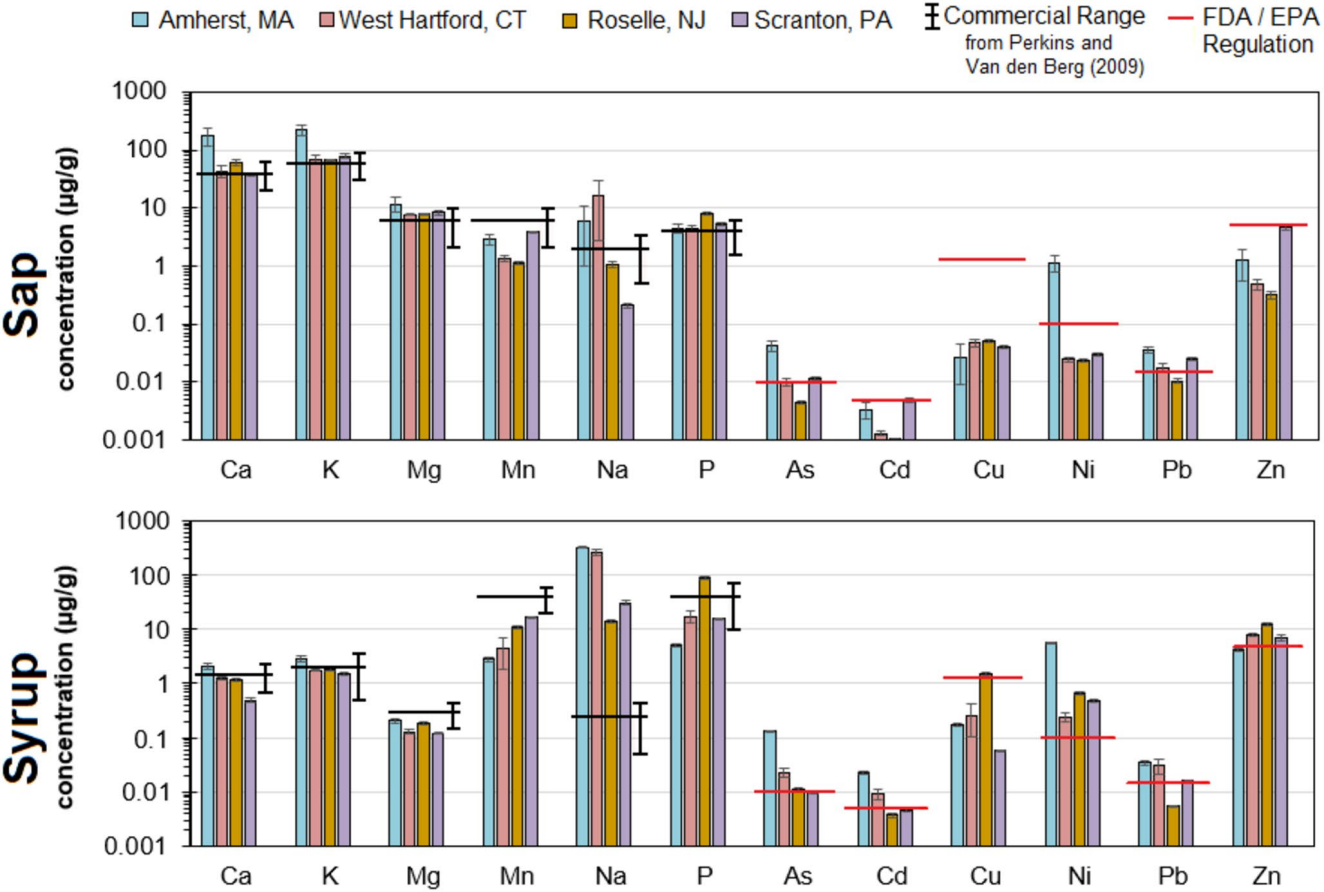
Nutrient and trace element concentrations in suburban soils, maple sap, and maple syrup tapped for syrup in this study. Soil properties are also provided. Commercial ranges are from Perkins and van den Berg (2009). Bars represent the mean concentration with standard error bars present. The red line denotes the US FDA regulatory limit for food

Maple syrup samples were collected from each of the four suburban artisanal producers following evaporation to syrup. While sap is a better assessment of element uptake by sugar maples, analysis of the syrup is most important as it represents concentrations reaching the consumer. Maple syrup concentrations of Al, Ca, K, Mg, Na, and P concentrations (Fig. 2) were within the range of concentrations measured in commercial maple syrup reported by Perkins and van den Berg (2009) and Mohammed et al. (2022). This finding is converse to the elevated sap concentrations measured but does not necessarily mean that maple sap nutrient concentrations were low. There is a wide range of nutrient concentrations measured in syrup, ranging 1 to 2 orders of magnitude, potentially due to variability in sap, filtration, settling, and evaporation material, and techniques employed by producers. In commercial operations, a typical ratio for volume reduction is 150 L of sap reducing down to 3.5 L of syrup yielding a syrup/sap concentration factors (CFs) of approximately 40. Using Ca, K, Mg, and Na as conservative tracers for the reduction from sap to syrup, the four cities studied had syrup/sap CFs of 16 to 24. It must be noted that Mn and P were not conserved and had a syrup/sap CFs that ranged from 1 to 11 with an average of 4 across the four cities. We hypothesize the loss of Mn and P is due to precipitation with particulates filtered from the final syrup produced. Trace elements in maple syrup from at least one suburban town exceeded FDA food/EPA drinking water regulatory limits (Fig. 2). Maple syrup As, Cd, Ni, and Zn concentrations from all four suburban towns met or exceeded the FDA food/EPA drinking water regulatory limits. Our maple syrup As and Cd concentrations were lower than those reported by Saraiva et al. (2022) but maple syrup Pb concentrations were comparable with other studies (Mohammed et al. 2022). Only Roselle, NJ maple syrup Cu concentrations exceeded FDA/EPA regulatory limits. Despite having the highest soil Pb concentrations, Roselle, NJ maple syrup Pb concentrations did not exceed the FDA/EPA regulatory limits while the other three suburban towns did. Our maple syrup trace element concentration data suggest that soil concentrations are not the dominant factor controlling sugar maple syrup trace element concentrations for many metals, such as Ni, Pb, and Cd. The uptake of Cd, Ni, and Pb at Roselle, NJ were likely decreased by the abundance of Ca, which may have competed for cation exchange and root uptake by the sugar maple and their mycorrhizal fungal symbionts. We leveraged syrup/sap ratios for each trace element to quantify the change in concentration from evaporating and processing maple sap to make maple syrup. Syrup/sap ratios were 1 to 3 for As, 1 to 7 for Cd, 2 to 29 for Cu, 5 to 16 for Ni, 0.5 to 1.7 for Pb and 2 to 38 for Zn. The syrup/sap ratios show that trace elements were generally enriched due to evaporation. Trace elements they may also be added to the syrup from interactions with metal surfaces of the equipment. At Roselle, NJ, syrup/sap CFs were 29 to 38 for Cu, Ni, and Zn, which exceeded syrup/sap CFs for the Ca, Mg, K, and Na salts of 13 to 29, indicating net addition of Cu, Ni, and Zn during the evaporation process most likely from their use of stainless steel vessels. Pb had the lowest syrup/sap CFs for all trace elements of 0.5 and 0.7, showing net loss of Pb during evaporation at these two sites. The Pb may have adsorbed onto surfaces or particles and removed from the syrup and a greater rates than other trace elements like As and Cd due to its lower solubility.

### Total Hazard Quotient

**Fig. 3:**
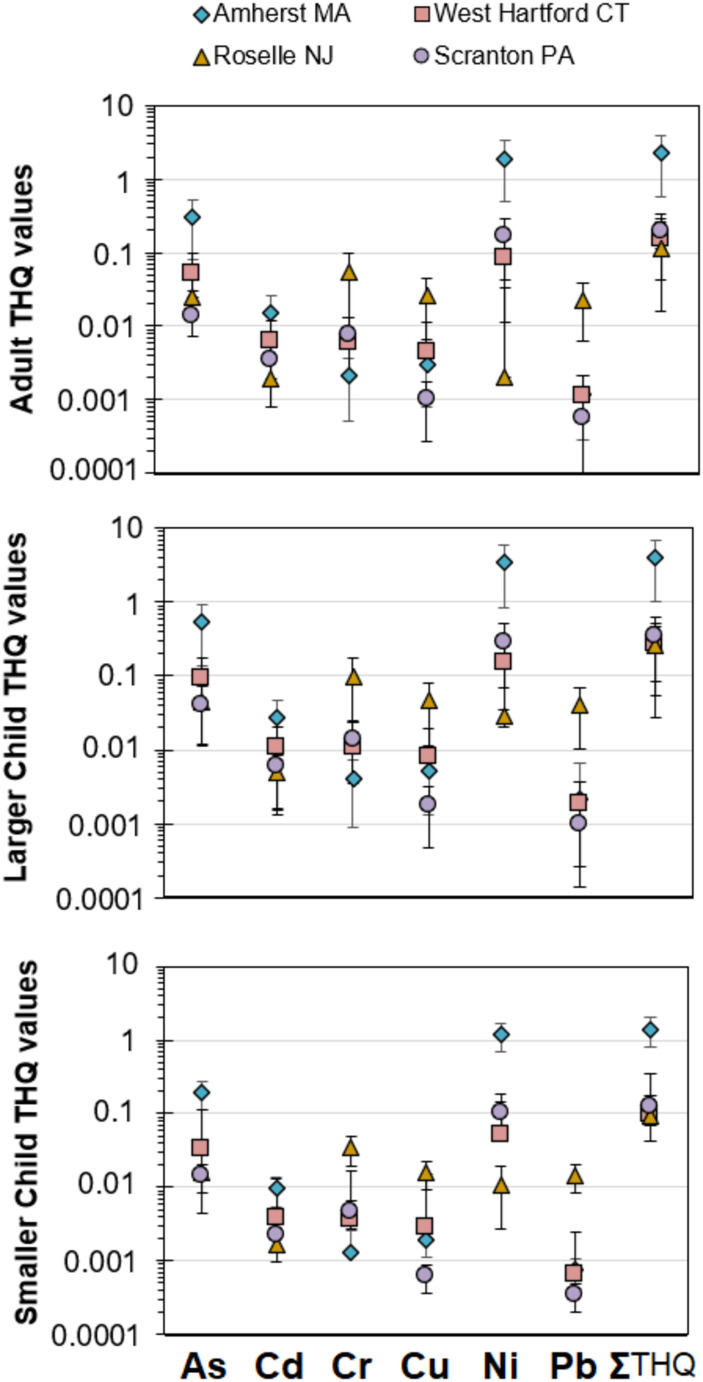
Total Hazard Quotients for non-carcinogenic health risk calculated for six of the potentially toxic trace elements in each of the four maple syrups for three different age groups: adults (80 kg body mass), large child (40 kg body mass), and small child (10 kg body mass). For the summed THQ, the six potentially toxic elements were summed. Zinc was omitted due to its lesser role in toxicity

The THQ assessment and Monte Carlo approach was used to estimate potential non-carcinogenic health risk from consumption of the four artisanal maple syrups produced from suburban sugar maple trees (Fig. 3). THQs were calculated for three age classes: adult (assumed to be 80 kg), larger child (12 years of age 40 kg), and smaller child (toddler 1–2 years of age, 10 kg). For adults, larger children, and small children, the THQs for the individual trace elements and summed THQ values were safe for consumption, even under high consumption rates (> 60 g/d for adults and larger children, > 4 g/d for smaller children). Only the Amherst maple syrups posed a non-carcinogenic hazard if consumed at high daily consumption rates, such as substituting maple syrup for most other forms of processed sugars. Since this is an unlikely situation, it can be safe to conclude these urban forest produced sugars are safe for typical consumption rates (< 20 g/d). To better understand how consumption rate affects THQ, we reverse calculated the daily consumption rate that would cause THQ to exceed 1.0 for each of the four artisanal maple syrups for an adult, larger child, and smaller child. Our results show that adults can consume > 60 g/d of the Amherst artisanal maple syrups for low non-carcinogenic health risk. Similarly, a larger child can consume > 60 g/day of all four artisanal maple syrups with low to no non-carcinogenic health risk. However, > 4 g/day of the Amherst, MA artisanal syrup for a smaller child could pose a non-carcinogenic health risk.

## Conclusions

The overarching goal of this study was to investigate trace elements in sugar maple sap and syrup produced in suburban areas across the northeastern United States and if elevated concentrations are driven by their abundance in surrounding soils. Our results show that soil pollution was elevated in suburban areas and exceeded EPA limits for safety in these areas. Trace element concentrations in soils did not correlate with higher sugar maple sap and syrup concentrations; Roselle, NJ had the highest soil Cd, Cr, Cu, Pb, and Zn concentrations of the three towns but had the lowest Cd and Pb concentrations in maple sap and syrup.

Sugar maple sap exceeded FDA regulatory limits for As, Cd, Ni, Pb, and Zn at one or two of the four suburban towns but sugar maple syrup exceeded FDA regulatory limits for all seven trace elements, with all four sites exceeding the limits for As, Cd, Ni, and Zn. The evaporative process increased metals 5x to 15x for Cd, Cr, Cu, Ni and Zn but only 1x to 2x for As and Pb, indicating minimum enrichment during sap to syrup evaporation. THQ values for all four artisanal maple syrups suggest there is low to no risk of non-carcinogenic health hazard for adults and larger children at typical (< 8 g/d) to high daily consumption rates (> 60 g/d). However, the Amherst, MA maple syrup poses a risk to adults and smaller children (10 kg) if they consume maple syrup high rates. Reversing the THQ equations, we find that consumption of > 60 g/d for adults and large children or > 4 g/d of the Amherst, MA maple syrup poses a health risk. While high maple syrup consumption is unlikely, Welsh and Figueroa (2017) found that small children (toddlers) consumed on average 90 g/d of sugars. If parents substituted high fructose corn syrup to locally sourced maple syrup for their child, this could pose a distinct health risk with respect to chronic trace element toxicity in the small child if concentrations are elevated, such as in the Amherst, MA artisanal maple syrup. Our findings suggest that soil concentration tests may not provide an accurate indication of concentrations within maple sap and syrup, and testing is needed to evaluate if an artisanal maple syrup production is safe or may pose a risk under heavy chronic consumption.
